# Enzymes flying under the radar: Cryptic METTL3 can persist in knockout cells

**DOI:** 10.1371/journal.pbio.3001717

**Published:** 2022-07-20

**Authors:** Sigrid Nachtergaele

**Affiliations:** Department of Molecular, Cellular, and Developmental Biology, Yale University, New Haven, Connecticut, United States of America

## Abstract

The presence of the RNA modification m6A in the mRNA of METTL3 knockout cells has long been a contentious point. This Primer explores the implications of a PLOS Biology study revealing that alternatively spliced, catalytically active METTL3 isoforms persist in cells previously thought to lack the enzyme.

## Teaser

The presence of m^6^A in mRNA of METTL3 knockout cells has long been a point of confusion. In this issue of *PLOS Biology*, Poh and colleagues reveal alternatively spliced, catalytically active METTL3 isoforms that persist in cells previously thought to lack the enzyme.

RNA molecules are modified with over 160 chemically diverse groups that can tune RNA structure, stability, and function. While RNA modifications were first studied primarily in abundant noncoding RNAs, we now know that mRNA also carries several of these chemical marks [1]. In particular, the discovery and characterization of *N*^6^-methyladenosine (m^6^A) in mRNA over the last decade has led to an explosion of work demonstrating its function in a variety of organisms and cell types [2]. This methyl mark is installed on mRNA by the METTL3-METTL14 complex [3] and can regulate mRNA splicing, export, translation, and decay. However, as numerous groups sought to dissect m^6^A function and regulation, they ran into the puzzling result that mRNA m^6^A persisted in some cell lines despite apparent complete knockout of the catalytic component METTL3.

For instance, 2 studies independently generated *Mettl3* knockout mouse embryonic stem cells (mESCs) and found loss of approximately 60% and approximately 99% of mRNA m^6^A, respectively [4,5]. The first study used CRISPR-Cas9-mediated knockout with a guide RNA targeting exon 2 (exon2 *Mettl3* KO mESC) [4], while the second used Cre-Lox recombination to excise exon 4 (exon4 *Mettl3* KO mESC) [5]. At the time, it was thought that the METTL3 binding partner, METTL14, might have sufficient m^6^A methyltransferase activity to compensate for METTL3 loss. This has since been shown not to be the case, resulting in the hypothesis that another yet-to-be identified methyltransferase was responsible for the remaining m^6^A. While additional m^6^A methyltransferases, such as METTL16 [6] and METTL5 [7], have since been identified, their activity on mRNA is thought to be limited. Even if these enzymes could partially compensate for METTL3 loss, how this could result in such high variability in the amount of remaining m^6^A was mysterious. Genetic knockout of *Mettl3* represented a powerful tool to study the role of m^6^A in a variety of biological systems. But the fact that the modification persisted to such different degrees was a significant complication in interpreting these experiments and called into question whether the observed phenotypes were, indeed, the result of loss of m^6^A in mRNA.

Poh and colleagues [8] set out to settle this critical question through comprehensive characterization of the cells in question, including exon2 *Mettl3* KO mESC and exon4 *Mettl3* KO mESC. These cell lines were generated when research on m^6^A in mRNA was in its infancy, so each study used different reagents and methods to measure m^6^A modification and METTL3 protein levels. To ensure that technical differences were not to blame, m^6^A abundance in mRNA was re-analyzed by mass spectrometry, confirming the dramatic differences described previously. However, careful examination of western blots using 2 different anti-METTL3 antibodies revealed the presence of additional METTL3 species that varied across cell lines.

**Fig 1:**
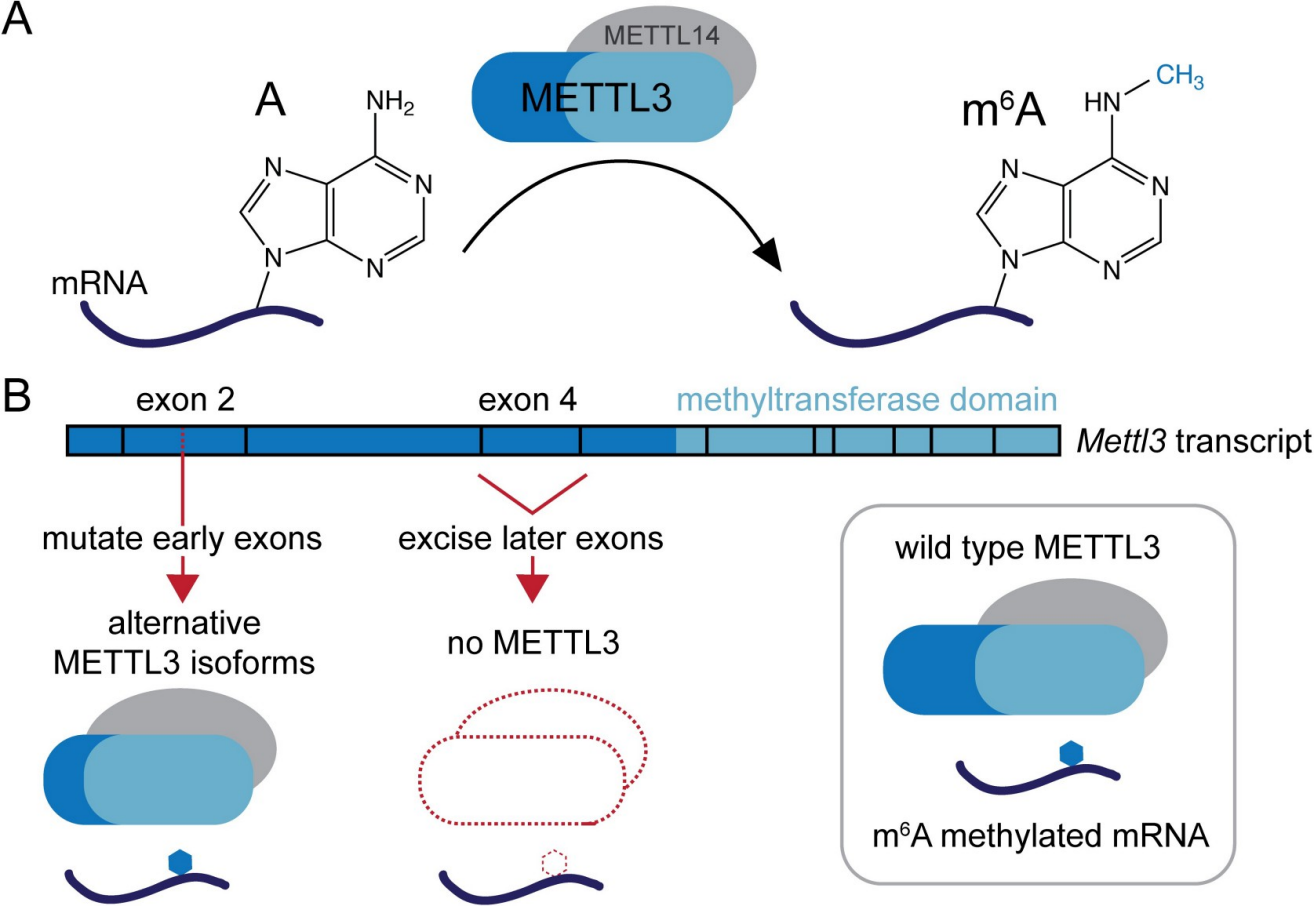
Alternative splicing generates functional METTL3 enzyme isoforms. (A) *N*^6^-methyladenosine (m^6^A) in mammalian mRNA is installed by a core complex containing METTL3 and METTL14 [3], as well as additional factors that are not shown. While METTL3 is the catalytic component, METTL14 is required for complex stability and activity. (B) The mouse *Mettl3* transcript contains multiple exons, two of which were targeted for mutagenesis to generate genetic knockouts in different studies. While excising exon 4 generated true *Mettl3* knockout mouse embryonic stem cells that lack m^6^A in mRNA [5], CRISPR-Cas9-mediated mutagenesis of exon 2 allowed for alternatively spliced *Mettl3* transcripts to be generated [4,8]. Some of these new transcript isoforms could be translated in cells, forming functional METTL3 enzyme. The discovery of these METTL3 isoforms finally explains why different cell lines previously thought to completely lack this enzyme show large variation in the amount of residual m^6^A in mRNA.

The fact that exon2 *Mettl3* KO mESCs had significantly more residual m^6^A than exon4 *Mettl3* KO mESCs, combined with the possible presence of alternative METTL3 protein isoforms, suggested that the different methods and genomic locations used for mutagenesis may have resulted in alternatively spliced transcripts (Fig 1). Of these transcripts, some could have translation start sites that would allow for catalytically active METTL3 isoforms to be translated. To test this hypothesis, Poh and colleagues first used 5′ RACE to identify which alternatively spliced *Mettl3* transcripts were present in each cell line. Using this information, they determined which METTL3 isoforms predicted to be translated from these transcripts had m^6^A methyltransferase activity. Polysome profiling then confirmed that these alternatively spliced *Mettl3* transcripts were indeed translated in cells. As a secondary demonstration that isoforms of METTL3 itself, and not another methyltransferase, were responsible for the residual m^6^A, Poh and colleagues took advantage of a recently developed METTL3 inhibitor, STM2457 [9]. Treatment of exon2 *Mettl3* KO mESC with this inhibitor significantly reduced mRNA m^6^A levels, indicating that the origin of this m^6^A was indeed a METTL3 isoform, as STM2457 does not inhibit other RNA methyltransferases.

While it remains possible that small numbers of mRNA m^6^A sites are installed by other RNA methyltransferases, the multiple orthogonal approaches taken in this work strongly suggest that the majority of this modification is installed by the METTL3-METTL14 complex. This work also provides new clarity on the question of whether *Mettl3* is an essential gene. *Mettl3* knock out in mice is embryonic lethal, and genetic screens in human cell lines suggest that they also do not survive *METTL3* loss. Delving into the DepMap dataset [10], Poh and colleagues found that the majority of cell lines were, indeed, dependent on *METTL3* for survival. The fact that stable, viable *METTL3* knockout human cell lines could be generated at all was therefore a paradox, but it now appears likely that many of these lines survived by relying on alternatively spliced *METTL3* isoforms. Taken together, this study provides an elegant answer to a question that has plagued the field since *METTL3* knockout cell lines and organisms were first generated. Moreover, it provides a framework within which researchers can now assess their own reagents and results.

More broadly, however, it should also serve as a cautionary tale. CRISPR-Cas9 has revolutionized our ability to quickly generate knockout cell lines for almost any gene of interest. But, as this work reveals, the details of its implementation can dramatically impact the results. The analysis by Poh and colleagues demonstrated that a smaller mutation in an earlier exon was insufficient to eliminate METTL3 activity, while excision of a later exon resulted in a true genetic knockout [8]. In this case, a combination of alternative splicing and the ability of individual protein domains to form stable folds came together to produce sufficient cryptic METTL3 activity to install significant amounts of m^6^A on mRNA. However, these 2 processes, alternative splicing and stable folding of protein domains, are of course ubiquitous in biology. Thus, it is likely that functionally incomplete knockout cell lines are more common than we realize. Moving forward, proactively thinking about possible alternative isoforms when designing knockout strategies, as well as more detailed characterization of the resulting knockout cell lines, will be critical to ensure correct data interpretation.
